# Medical Appointments in Philadelphia

**Published:** 1848-05

**Authors:** 


### MEDICAL APPOINTMENTS IN PHILADELPHIA.

Thomas F. Betton, M. D., of Germantown, Pennsylvania, Professor of Surgery in the Franklin Medical College, vice C. C. Vanwyck, M. D., resigned. George Fox, M. D., Surgeon to the Pennsylvania Hospital, in the place of Dr. Jacob Randolph, deceased.

